# A Biomimetic Adhesive Approach for Restoring a Cracked Tooth in a Single Visit: A Case Report

**DOI:** 10.1155/crid/5525524

**Published:** 2026-02-27

**Authors:** Zhenxiang Lin, Minrui Xu, Ruizhen Chen, Jie Lin

**Affiliations:** ^1^ Department of Stomatology, Fujian Provincial Governmental Hospital, Fuzhou, Fujian, China; ^2^ Department of VIP Dental Service, Fujian Key Laboratory of Oral Diseases, School and Hospital of Stomatology, Fujian Medical University, Fuzhou, Fujian, China, fjmu.edu.cn; ^3^ Department of Crown and Bridge, School of Life Dentistry at Tokyo, The Nippon Dental University, Chiyoda-ku, Tokyo, Japan, ndu.ac.jp

**Keywords:** biomimetic restorative dentistry, case report, cracked tooth, morphology-driven preparation technique, single visit

## Abstract

**Objective:**

This report evaluates the morphology‐driven preparation technique (MDPT) for biomimetic restoration of a cracked maxillary first molar, focusing on its tooth‐preserving benefits and clinical outcomes.

**Case Presentation:**

A 36‐year‐old male presented with 2 months of masticatory pain and recent spontaneous pain in the upper right posterior region (Tooth 16). Examination revealed occlusal cracks, confirmed as cracked tooth syndrome radiographically. Treatment included microscopic root canal therapy (biomechanical preparation, irrigation, and hybrid obturation) followed by MDPT‐based restoration. The minimally invasive approach preserved natural morphology using digital impressions (3Shape TRIOS 5) and placed a lithium disilicate veneer with dual‐cure resin cement under optimized bonding protocols.

**Results:**

MDPT achieved a complete pain resolution and functional restoration while preserving maximum dentin structure. Minimal preparation and immediate dentin sealing enhanced stability and reduced microleakage risk, with excellent esthetic integration.

**Conclusion:**

MDPT represents an effective biomimetic solution for cracked teeth, combining minimal invasiveness with optimal clinical outcomes. The technique demonstrates significant advantages in tooth structure preservation and restoration longevity. Comparative studies are needed to further validate its clinical benefits.

## 1. Introduction

Cracked tooth syndrome poses a diagnostic and restorative challenge due to its subtle clinical presentation and the risk of further structural compromise, driving the need for minimally invasive yet durable restorative approaches. One such approach is the morphology‐driven preparation technique (MDPT) [1], which emphasizes using the natural tooth morphology to guide preparation design. This technique adopts a minimally invasive approach by customizing the preparation to the tooth′s anatomical characteristics and functional needs, thereby preserving as much healthy tooth structure as possible [2, 3].

MDPT is especially beneficial in cases requiring indirect restorations, such as ceramic crowns, onlays, and veneers, where precise preparation is crucial for achieving an accurate fit and ensuring long‐term success [4]. Unlike traditional preparation methods, which typically follow a uniform reduction pattern, MDPT takes into account the unique morphology of each tooth. This allows for a more conservative removal of tooth structure and enhanced biomechanical performance [5–7]. By focusing on the individual characteristics of each tooth, this patient‐centered approach not only preserves the tooth′s structural integrity but also improves esthetic outcomes and reduces the risk of postoperative complications [8–10].

Cracked tooth syndrome is a common clinical challenge that often presents with pain during mastication [11, 12]. In recent years, with advancements in dental bonding and material science, many research studies have reported the use of occlusal veneers to treat cracked teeth [13]. Accurate diagnosis and appropriate restorative strategies are crucial to prevent further structural compromise and alleviate symptoms. MDPT offers a conservative and precise approach to preparing teeth for restorations while preserving maximum tooth structure. This case report describes the diagnosis and management of a patient presenting with a cracked maxillary first molar, treated using MDPT and a porcelain laminate veneer restoration.

## 2. Case Presentation

A 36‐year‐old male patient presented with a 2‐month history of pain in the upper right posterior tooth (Tooth 16) when biting down on hard objects, accompanied by recent onset of nocturnal and spontaneous pain for 1 day. Initially, the patient did not seek treatment, but as the pain worsened, he decided to consult a dental professional. Due to imminent relocation for work, the patient specifically requested completion of treatment in a single visit whenever possible. The patient was otherwise in good general health, with no known history of systemic diseases, including leukemia, liver disease, cardiovascular issues, hypertension, or diabetes. Additionally, he denied any history of infectious diseases, allergies, trauma, previous surgeries, or exposure to toxins.

The patient provided written informed consent for the publication of this case report and any accompanying images. The treatment procedures were conducted in accordance with the Declaration of Helsinki, and the protocol for reporting this case was reviewed and approved by the Ethics Committee of the Fujian Provincial Governmental Hospital (Approval No.: RL2024‐02).

The initial clinical condition is presented in Figure 1, which shows the baseline intraoral photographs documenting the patient′s occlusal relationship and tooth morphology. Upon clinical examination, Tooth 16 showed no response to percussion or probing, and a positive response to cold testing (+). Visible cracks were observed on the occlusal surface, but there was no evidence of gingival inflammation or tooth mobility. Radiographic findings from a periapical radiograph revealed no significant low‐density areas at the root apex of Tooth 16. The diagnosis was a cracked tooth syndrome with reversible pulpitis affecting Tooth #16. Based on the American Association of Endodontists (AAE) classification, the crack was identified as extending into the pulp chamber but not involving the pulpal floor upon microscopic examination, warranting endodontic treatment followed by a restoration. The proposed treatment plan included root canal therapy, followed by restoration of Tooth 16 using a porcelain laminate veneer, utilizing the MDPT.

Figure 1Initial intraoral photographs. (a) Right lateral occlusal view showing the cracked tooth; (b) left lateral occlusal view demonstrating the previously endodontically treated and crowned first molar (cracked 1 year prior); (c) occlusal view of the right posterior teeth; (d) methylene blue staining clearly revealing the crack line on the maxillary right first molar.

**Figure:**
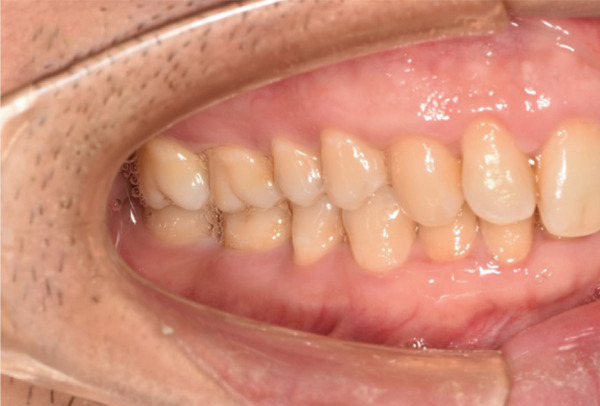
(a)

**Figure:**
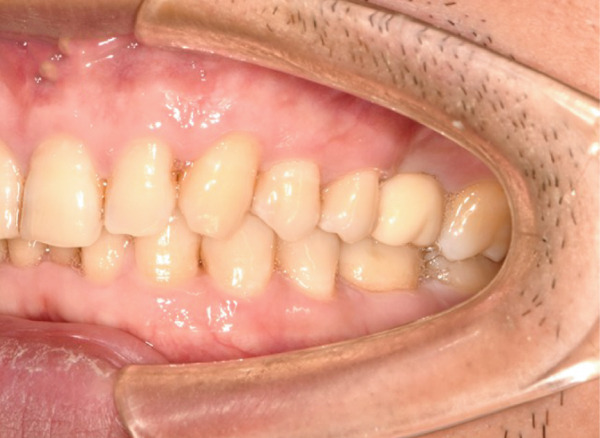
(b)

**Figure:**
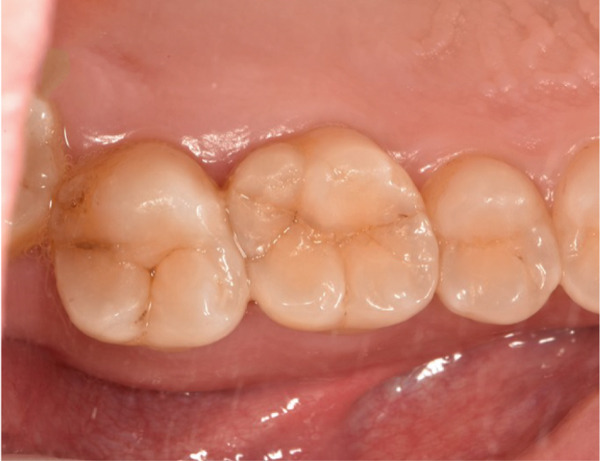
(c)

**Figure:**
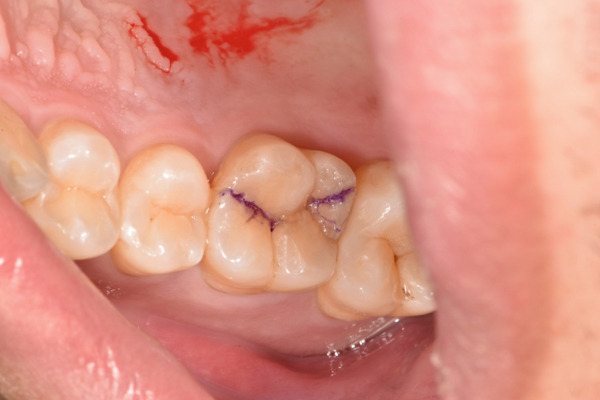
(d)

### 2.1. Restorative Phase: MDPT Protocol

The restoration of a cracked, root canal‐treated maxillary first molar using the MDPT involves a precise and minimally invasive approach. The goal is to preserve a healthy tooth structure while ensuring optimal adhesion and biomechanical support for the lithium disilicate IPS e.max Press (Ivoclar Vivadent AG, Liechtenstein) restoration. The preparation focuses on butt‐joint margins (1.2–1.3 mm) in the proximal boxes and inclined planes (hollow chamfer) on the buccal and palatal walls, maintaining closed proximal contacts.

### 2.2. Initial Evaluation and Preparation

Before beginning the preparation, the tooth is assessed for crack extent, remaining dentin thickness, and periodontal health. A provisional restoration may be placed if the tooth requires stabilization before final restoration. The tooth preparation process and digital workflow are illustrated in Figure 2, which includes (a) minimally invasive preparation following MDPT principles, (b) the intraoral scanning procedure for digital impression acquisition, and (c) occlusal registration captured through the intraoral scanning system.

Figure 2Tooth preparation and intraoral scanning. (a) Tooth preparation following MDPT principles; (b) intraoral scanning procedure; (c) occlusal registration obtained through intraoral scanning.

**Figure:**
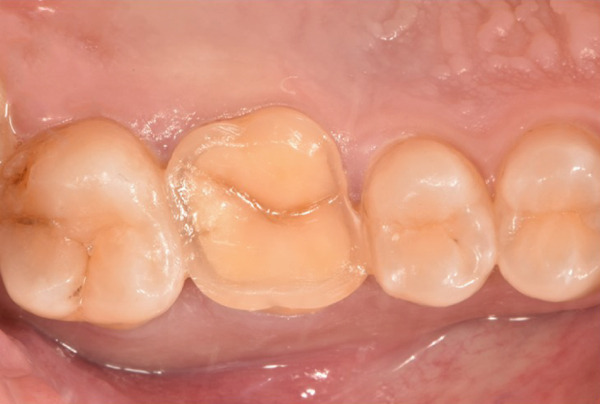
(a)

**Figure:**
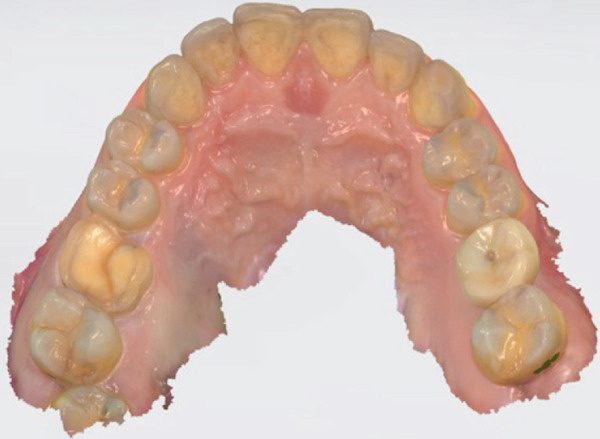
(b)

**Figure:**
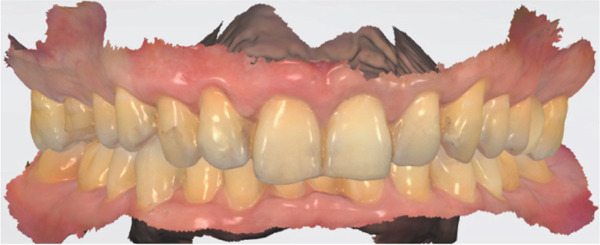
(c)

### 2.3. Proximal Box Preparation (Butt‐Joint Margins)

Following the successful administration of local anesthesia (articaine 1:100,000 epinephrine infiltration at Tooth #16), the proximal box preparations were initiated using a tapered cylindrical diamond bur (medium grit, 1.2‐mm tip diameter). The proximal walls were reduced to a precise depth of 1.2–1.3 mm, establishing well‐defined 90° cavosurface margins without beveling. Internal line angles were carefully rounded to reduce stress concentration, whereas the natural proximal contact areas were meticulously preserved.

### 2.4. Occlusal Reduction

The occlusal surface is reduced anatomically using a rounded‐end diamond bur, following the natural fissure pattern. A uniform reduction of 1.0–1.5 mm is maintained, guided by a silicone index from the diagnostic wax‐up. Sharp angles are eliminated, and smooth transitions are created between the cusps and proximal boxes to ensure even stress distribution.

### 2.5. Buccal and Palatal Axial Walls (Inclined Planes—Hollow Chamfer)

The buccal and palatal walls are prepared based on their position relative to the maximum contour line (equator) of the tooth. For areas coronal to the equator, a hollow chamfer (concave bevel) is created using a chamfer bur, maintaining a 20°–30° inclination to optimize enamel prism exposure for bonding. The bur is used only at the tip to achieve a smooth, continuous margin. If the axial wall extends apical to the equator, a butt‐joint margin is prepared instead. The transition between the proximal boxes and axial walls is carefully blended with curved “slip roads” to avoid sharp edges.

### 2.6. Finishing and Verification

After the preparation, all margins are refined with fine‐grit diamond burs and polished with rubber wheels to ensure smooth, well‐defined edges. The final preparation is checked for uniform thickness (1.2–1.5 mm minimum), absence of undercuts (using a silicone index), and proper marginal continuity.

### 2.7. Impression and Laboratory Communication

A digital impression was then taken using the 3Shape TRIOS 5 intraoral scanner. Specifying the margin design (butt‐joint proximally, hollow chamfer axially) and requesting a monolithic e.max Press restoration with a minimum thickness of 1.2 mm. The shade 2R2.5 is selected under neutral lighting using the VITA 3D‐Master guide. During root canal treatment, the technician performs chairside fabrication of the restoration.

### 2.8. Root Canal Treatment Procedure

Absolute isolation was achieved using a latex rubber dam system, Hygienic Dental Dam (Coltene/Whaledent AG, Switzerland), secured to adjacent teeth. The initial access cavity preparation was performed with a 1.5‐mm reduction using a high‐speed diamond fissure bur Brasseler 856‐014 (Brasseler USA, United States) with water cooling to access the pulp chamber, followed by refinement with ultrasonic tips (P5 XS Newtron, Satelec, France) to fully expose the pulp chamber. The root canal orifices were carefully identified under 16× magnification (OMS2380, Zumax Medical Co. Ltd., Suzhou, China) using DG‐16 endodontic explorers. Microscopic examination after access cavity preparation and chamber debridement revealed no extension of the tooth crack to the pulpal floor.

The electronic apex locator (Raypex 6, VDW GmbH, Munich, Germany) measurements, cross‐verified with parallel periapical radiographs, established the precise working lengths: MB‐16, MB2‐16, DB‐17, and P‐19 mm. The root canals were instrumented using a thermally treated nickel–titanium reciprocating file (Reciproc Blue R25/0.06; VDW GmbH, Munich, Germany) according to the manufacturer′s recommended motion parameters (150° counterclockwise/30° clockwise rotation). Throughout the preparation process, 17% EDTA gel (VDW Clean) was applied as a lubricant. The irrigation protocol alternated between 0.5% NaOCl (5 mL/canal) and 17% EDTA (3 mL/canal), enhanced by ultrasonic activation (Irrisafe Satelec tips, 30 s per canal at 30 kHz).

Post‐instrumentation canal drying was achieved using sterile absorbent points (Dentsply Sirona), followed by verification of master cone adaptation (25/0.06 GP cone tug‐back at 0.5 mm from the radiographic apex). The obturation phase employed a hybrid technique: a preheated System B plugger (200°C ± 5°C) was used to deliver thermoplasticized gutta‐percha (Dentsply Thermafil), forming a 4‐mm apical seal. The root canal obturation was performed using an epoxy resin‐based sealer (AH Plus, Dentsply Sirona, Konstanz, Germany) according to the manufacturer′s instructions. Buchanan hand spreaders were used for incremental backfilling, achieving a homogeneous radiodensity on immediate postoperative radiographs. The sequential endodontic treatment procedures are documented in Figure 3, demonstrating (a) manual instrumentation following access cavity preparation using K‐files, (b) nickel–titanium rotary instrumentation for canal shaping, (c) radiographic verification of master cone fit at the determined working length, and (d) postoperative radiograph confirming three‐dimensional obturation with gutta‐percha sealer.

Figure 3Endodontic treatment procedures. (a) Manual instrumentation after access cavity preparation; (b) rotary instrumentation during root canal preparation; (c) working length determination radiograph with master cone; (d) post‐obturation radiograph showing completed root canal filling.

**Figure:**
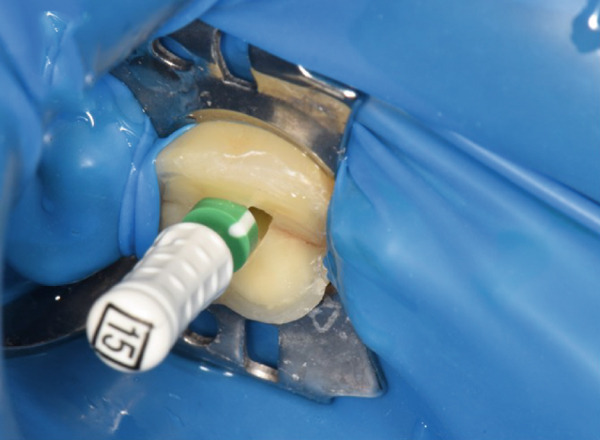
(a)

**Figure:**
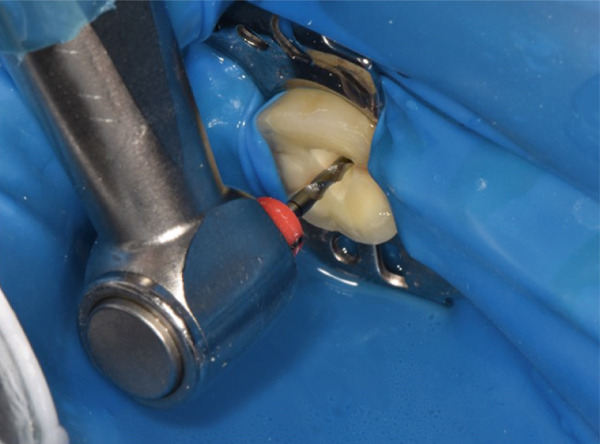
(b)

**Figure:**
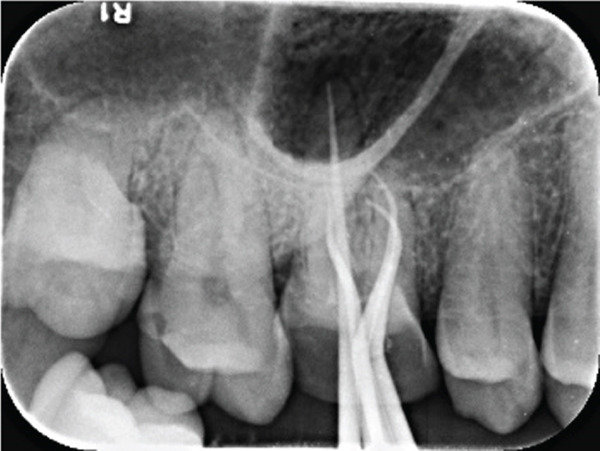
(c)

**Figure:**
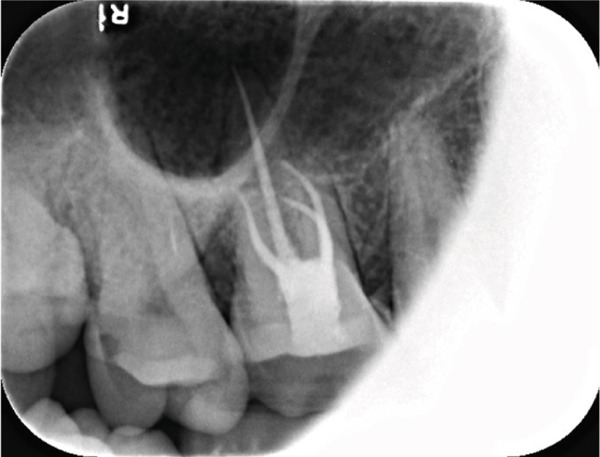
(d)

### 2.9. Cementation Protocol

The pretreatment protocol for the e.max Press restoration involved sequential surface conditioning: the intaglio surface was etched with 5% hydrofluoric acid for 20 s followed by a 90‐s application of silane coupling agent (Monobond Plus). After rubber dam isolation and 37% phosphoric acid etching of enamel bonding surfaces for 30 s, Single Bond Universal Adhesive (3M ESPE) was applied to exposed enamel and dentin, covered with a thin layer of flowable composite that was light‐cured for 20 s at 1200 mW/cm^2^, and then the access cavity was restored with short‐fiber–reinforced composite resin filled to 1 mm below the occlusal surface of the access opening and light‐cured for 20 seconds at 1200 mW/cm^2^ to facilitate direct bonding with the e.max restoration during final cementation. Dual‐cure resin cementation using RelyX Ultimate (3M ESPE) was performed under finger pressure after uniform adhesive application. Excess cement was meticulously removed prior to sectional light curing (20 s per buccal/lingual quadrant using a polywave LED unit). The final polishing protocol employed Sof‐Lex discs (coarse to superfine grits) to achieve enamel‐like surface gloss.

The entire procedure strictly adhered to minimally invasive principles. Both the endodontic treatment and restorative phases were performed under rubber dam isolation with microscopic assistance using a ZEISS OPMI PROergo operating microscope, successfully achieving the dual objectives of functional rehabilitation and biomechanical preservation.

Following the successful trial placement of the lithium disilicate veneer (IPS e.max CAD), functional and esthetic parameters were systematically verified: occlusal contacts were evenly distributed (detected using 8‐*μ*m articulating paper), marginal adaptation demonstrated < 40‐*μ*m discrepancy (evaluated under 20× microscopic magnification), and proximal contact tightness was optimized to allow dental floss passage with moderate resistance.

The restorative phase is presented in Figure 4, showing (a,b,c) the digital design process for the occlusal veneer restoration using CAD software, (d) the lithium disilicate (IPS e.max Press) restoration prior to crystallization, and (e,f) the definitively cemented restoration demonstrating optimal marginal integrity and functional occlusal contacts verified with 8‐*μ*m articulating paper. Clinical and radiographic evaluation at a 15‐month follow‐up demonstrated optimal restoration integrity, preserved tooth structure, and healthy periodontal conditions (Figure 5).

Figure 4Final restoration design and cementation. (a–c) Occlusal veneer restoration design process; (d) definitive lithium disilicate restoration; (e,f) cemented restoration showing proper marginal adaptation and occlusion.

**Figure:**
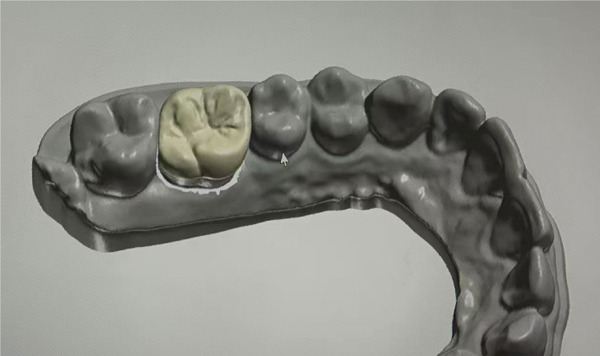
(a)

**Figure:**
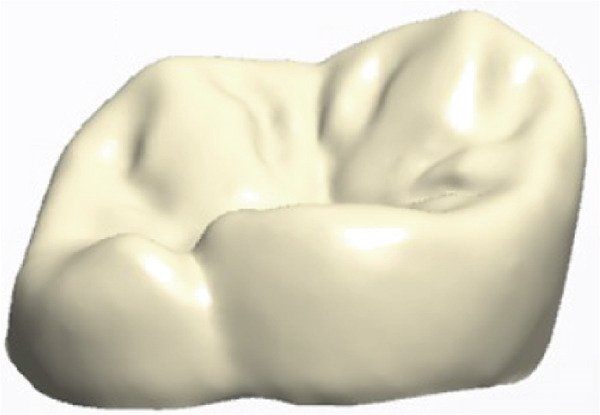
(b)

**Figure:**
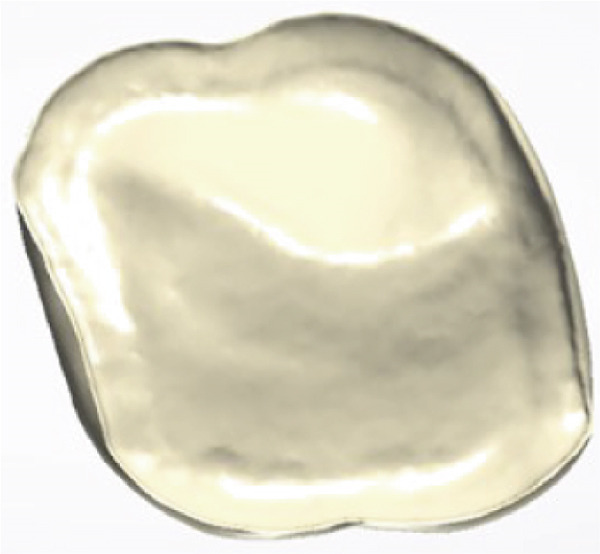
(c)

**Figure:**
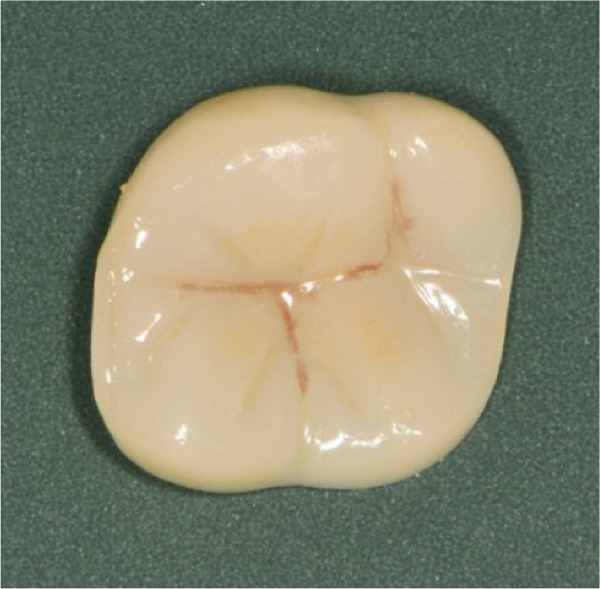
(d)

**Figure:**
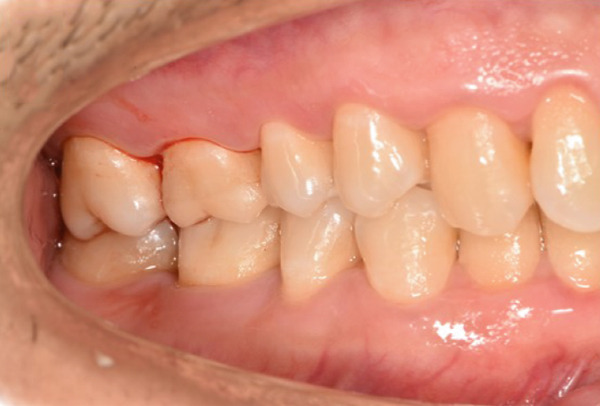
(e)

**Figure:**
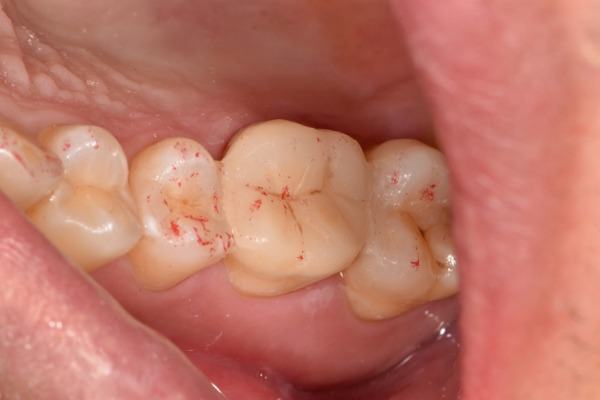
(f)

Figure 5A 15‐month follow‐up examination. (a–b) Occlusal and buccal views demonstrating excellent restoration integrity and healthy periodontal tissues; (c) follow‐up periapical radiograph confirming normal periapical and periodontal status.

**Figure:**
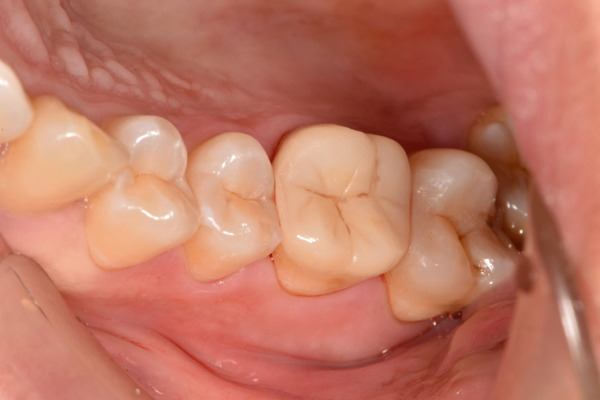
(a)

**Figure:**
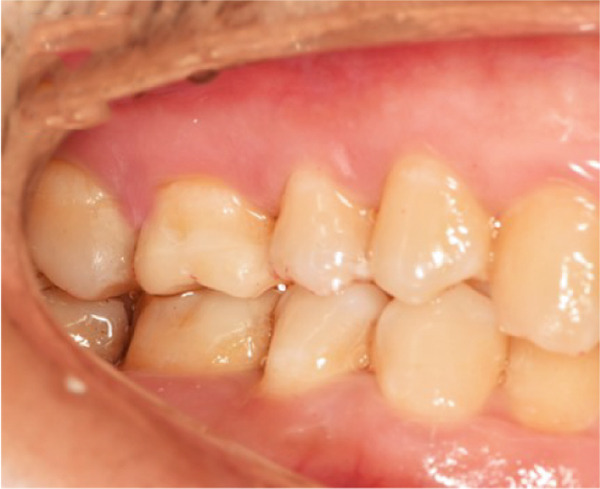
(b)

**Figure:**
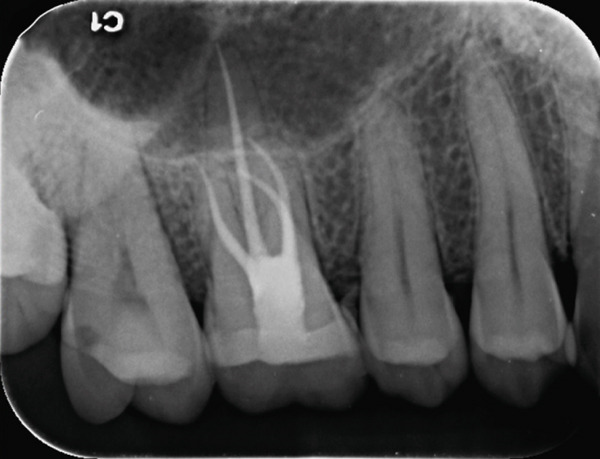
(c)

## 3. Discussion

Cracked tooth syndrome is a multifactorial condition whose etiology stems from the interplay of predisposing factors and triggering events, leading to the propagation of cracks within the tooth structure [14, 15]. A primary risk factor is the presence of parafunctional habits, such as bruxism or clenching, which subject teeth to prolonged and excessive occlusal forces beyond their physiological tolerance [16]. These forces are often concentrated on pre‐existing structural weaknesses, including large or failing restorations that compromise cusp integrity, deep developmental grooves, or endodontically treated teeth with reduced moisture content and resilience. Furthermore, occlusal factors like premature contacts, steep cuspal inclines, and malocclusion can create unfavorable stress distribution, acting as chronic triggers for crack initiation and propagation [17]. Age‐related physiological changes also contribute, as dentin becomes less elastic and more brittle over time, reducing the tooth′s ability to absorb masticatory shocks [18]. In the present case, although the patient did not report a history of bruxism, the combination of the tooth′s complex occlusal morphology and the likely presence of chronic, undetected occlusal overload could have been the inciting factors. Understanding this etiological background underscores the imperative for restorative strategies that not only address the existing defect but also mitigate future risk by reinforcing the tooth structure and ensuring harmonious occlusion, principles that are central to the biomimetic, morphology‐driven approach employed here. Many studies have demonstrated the effectiveness of veneers in treating cracked teeth [19–21]. In this case, the patient exhibited typical symptoms, including pain during mastication, and visible cracks were observed on the occlusal surface of Tooth 16.

The decision for single‐visit treatment was primarily patient‐driven due to imminent relocation, which aligns with the increasing emphasis on patient‐centered care in contemporary dentistry [22]. Importantly, pulpal inflammation was clinically diagnosed, and periapical radiographs revealed no evidence of periapical involvement. This combination of findings indicated that the pathology was confined to the root canal system, making single‐visit treatment biologically appropriate [23]. Immediate restoration following endodontic treatment enhances the adhesive interface quality by preventing interim contamination [24]. Furthermore, for cracked teeth, prompt definitive restoration is crucial to prevent crack propagation, as demonstrated by de Toubes et al. [25] in their long‐term study of bonded restorations for cracked teeth.

The selection of an occlusal veneer rather than full‐coverage crown was based on substantial evidence supporting minimally invasive approaches. Magne et al. [26, 27] demonstrated that occlusal veneers with a 1.0–1.5‐mm reduction preserve significantly more tooth structure compared with traditional crown preparations. This is particularly critical for endodontically treated teeth where preservation of remaining tooth structure is paramount for fracture resistance [28]. The MDPT protocol′s butt‐joint proximal design (1.2–1.3 mm) [1, 3] and hollow chamfer axial preparation further optimize structural preservation while ensuring adequate material thickness for lithium disilicate restorations.

MDPT was selected due to its ability to preserve the maximum amount of healthy tooth structure while ensuring precise and predictable restoration. Traditional preparation techniques often require uniform tooth reduction, which can weaken the remaining tooth structure. In contrast, MDPT is guided by the natural anatomical contours of the tooth, allowing for more conservative tissue removal and improved biomechanical integrity.

Lithium disilicate (e.max Press) was preferred over zirconia for several evidence‐based reasons. Its superior optical properties and ability to mimic natural tooth translucency were essential for this esthetically visible area [29]. Although zirconia offers greater flexural strength, lithium disilicate′s fracture toughness (2.75 MPa·m^1^/^2^) is more than adequate for occlusal veneers in premolars and molars, as shown by Marsico et al. [30]. Additionally, the adhesive bonding potential of lithium disilicate through hydrofluoric acid etching and silanization creates a monoblock effect, particularly beneficial for cracked teeth [31].

The use of SFRC for access cavity restoration represents an innovative approach combining endodontic and restorative advantages [32]. According to Soto‐Cadena et al. [33], SFRC provides superior fracture resistance compared with conventional composites, crucial for protecting the endodontically treated tooth. Its modulus of elasticity (12–15 GPa) better approximates dentin (18 GPa) than conventional composites, reducing stress concentration at the tooth‐restoration interface [34]. The 1‐mm sub‐occlusal placement allowed for optimal ceramic support while maintaining the adhesive interface integrity.

Additionally, the use of digital intraoral scanning ensures highly accurate impressions, improving the fit and esthetics of the final restoration. In this case, a porcelain laminate veneer was chosen for its excellent esthetic properties and durability. The final restoration demonstrated a proper marginal adaptation, accurate shade matching, and successful alleviation of the patient′s symptoms.

Future research should explore the long‐term clinical outcomes of MDPT across diverse patient populations and tooth types. Additionally, comparative studies between MDPT and conventional preparation methods would further validate its effectiveness in preserving tooth integrity and minimizing complications.

## 4. Conclusion

This case illustrates how the MDPT principles combined with contemporary materials and digital workflows can successfully address cracked tooth syndrome while maximizing tooth structure preservation. The treatment approach aligns with current trends in minimally invasive dentistry and demonstrates excellent short‐term outcomes. Long‐term follow‐up would be valuable to further validate this protocol′s durability, particularly regarding the SFRC‐ceramic interface performance under functional loading.

**Nomenclature**
MDPT: morphology‐driven preparation technique

## Author Contributions

All authors contributed to the conception and design of the case report. The initial draft of the manuscript was written by Z.L. and J.L., with all authors providing feedback on earlier versions. Z.L. was responsible for the clinical diagnosis and treatment of the case. M.X. and R.C. handled the methodology, original draft preparation, visualization, and investigation. Z.L. and J.L. contributed to the original draft preparation, data curation, and investigation. Additionally, Z.L. and J.L. provided supervision, conceptualization, validation, and manuscript review.

## Funding

This study was supported by Fujian Provincial Department of Science and Technology Guiding Project (2024Y0034). The authors declare that the funding bodies had no role in the design of the study, the collection, analysis, and interpretation of data, or in writing the manuscript.

## Ethics Statement

The protocol was approved by the Research Ethics Committee of Fujian Provincial Governmental Hospital.

## Consent

Written informed consent was obtained from the patient for the publication of this case report and the accompanying clinical images.

## Conflicts of Interest

The authors declare no conflicts of interest.

## Data Availability

All data generated or analyzed during this study are included in this article. Further enquiries can be directed to the corresponding author (Jie Lin, Email: linjie.dds@gmail.com).
